# Three dimensional delayed enhancement cardiovascular magnetic resonance imaging of the left atrium and pulmonary veins for atrial fibrillation ablation

**DOI:** 10.1186/1532-429X-15-S1-T5

**Published:** 2013-01-30

**Authors:** Rick Wage, Lay Koon Tan

**Affiliations:** 1 CMR, Royal Brompton Hospital, London, UK; 2 Cardiology, National Heart Institute, Kuala Lumpur, Malaysia

## Background

Atrial fibrillation (AF) is the commonest sustained arrhythmia afflicting the adult population. Identifying and ablating triggers in the left atrium (LA) or more often the pulmonary veins (PV) with radiofrequency energy to restore sinus rhythm is fast becoming the mainstay of treatment. The success of the procedure often requires a combination of imaging techniques with an electroanatomical mapping system. We describe the use of three dimensional (3D) delayed enhancement cardiovascular magnetic resonance imaging (DE-MRI) to provide a road map of the anatomy as well as regions of scar tissues in the LA and PV for AF ablation.

## Methods

Images were acquired with a Siemens Avanto 1.5 tesla scanner. Standard acquisition of cardiac anatomy and function were performed as well as the atrial short-tau inversion recovery (STIR) sequences in the axial plane. In addition, 3D steady-state free precession (SSFP) axial plane, 3D magnetic resonance angiography (MRA) coronal plane and 3D DE-MRI axial plane of both LA and PV were acquired.

## Results

The 3D SSFP axial plane, 3D MRA coronal plane and 3D DE-MRI axial plane of both LA and PV obtained were then incorporated into an electroanatomical mapping system which generated a 3D road map of the LA and PV together with regions of scar tissues as shown by DE-MRI. The merging of this road map with the electroanatomical map enables AF ablation to be done effectively.

## Conclusions

Together with the electroanatomical mapping system, 3D DE-MRI provides an invaluable non-invasive assessment of the anatomy and scar tissues of the LA and PV for AF ablation.

## Funding

No external funding.

